# Human neural progenitors express functional lysophospholipid receptors that regulate cell growth and morphology

**DOI:** 10.1186/1471-2202-9-118

**Published:** 2008-12-11

**Authors:** Jillian H Hurst, Jennifer Mumaw, David W Machacek, Carla Sturkie, Phillip Callihan, Steve L Stice, Shelley B Hooks

**Affiliations:** 1 Department of Pharmaceutical and Biomedical Sciences, University of Georgia, Athens, GA, USA; 2 Regenerative Bioscience Center Animal Science Department, University of Georgia, Athens, GA, USA; 3 Aruna Biomedical, Inc., Athens, GA, USA; 4 377 Wilson Pharmacy Building, 250 West Green Street, Athens, GA 30602-2352, USA

## Abstract

**Background:**

Lysophospholipids regulate the morphology and growth of neurons, neural cell lines, and neural progenitors. A stable human neural progenitor cell line is not currently available in which to study the role of lysophospholipids in human neural development. We recently established a stable, adherent human embryonic stem cell-derived neuroepithelial (hES-NEP) cell line which recapitulates morphological and phenotypic features of neural progenitor cells isolated from fetal tissue. The goal of this study was to determine if hES-NEP cells express functional lysophospholipid receptors, and if activation of these receptors mediates cellular responses critical for neural development.

**Results:**

Our results demonstrate that Lysophosphatidic Acid (LPA) and Sphingosine-1-phosphate (S1P) receptors are functionally expressed in hES-NEP cells and are coupled to multiple cellular signaling pathways. We have shown that transcript levels for S1P1 receptor increased significantly in the transition from embryonic stem cell to hES-NEP. hES-NEP cells express LPA and S1P receptors coupled to G_i/o _G-proteins that inhibit adenylyl cyclase and to G_q_-like phospholipase C activity. LPA and S1P also induce p44/42 ERK MAP kinase phosphorylation in these cells and stimulate cell proliferation via G_i/o _coupled receptors in an Epidermal Growth Factor Receptor (EGFR)- and ERK-dependent pathway. In contrast, LPA and S1P stimulate transient cell rounding and aggregation that is independent of EGFR and ERK, but dependent on the Rho effector p160 ROCK.

**Conclusion:**

Thus, lysophospholipids regulate neural progenitor growth and morphology through distinct mechanisms. These findings establish human ES cell-derived NEP cells as a model system for studying the role of lysophospholipids in neural progenitors.

## Background

We have previously generated a stable neuroepithelial (NEP) cell line derived from human embryonic stem (hES) cells (hES-NEP) that is grown under adherent conditions, is self-renewing, and stably maintains capacity for neuronal or glial differentiation. These hES-NEP cells recapitulate morphological and phenotypic features of neural progenitor cells isolated from fetal tissue [1]. Such a cell line has potential both as a source for specific neuronal lineages to be used in hES cell neural therapy and as an *in vitro *model system in which to study human NEP cell function and its regulation by signaling mediators such as lysophospholipids. The lysophospholipid signaling mediators Lysophosphatidic Acid (LPA) and Sphingosine 1-phosphate (S1P) are critical regulators of neural development, modulating neural growth, morphogenesis, and differentiation.

Lysophospholipid signaling has been implicated in mediating diverse physiological and pathological responses, including cancer progression, wound healing, angiogenesis, cardiovascular development, and, more recently, neural development (Reviews: [2-5]). There is strong evidence that both LPA and S1P are critical in early neural development, as mouse embryos that lack enzymes for S1P or LPA synthesis exhibit severe neural tube defects. Specifically, mice with genetic deletion of Sphingosine kinases required for production of S1P developed cranial neural tube defects as a result of increased apoptosis, decreased mitosis and subsequent thinning of the neuroepithelial progenitor cell layer [6]. These data suggest that S1P mediates anti-apoptotic and pro-growth signaling in normal neuroepithelial development. Similarly, genetic deletion of Autotaxin, the enzyme responsible for production of LPA in the brain, yields embryonically lethal mice with neural tube defects. In these embryos, the neural tube fails to close completely and is kinked [7]. Further, embryos lacking LPA exhibited asymmetric neural headfold, reflecting large effusions with high levels of apoptotic cells [8]. These studies demonstrate critical and distinct roles of S1P and LPA in early neural development.

LPA and S1P receptors are expressed in neural progenitors, neurons, and oligodendrocytes in the developing and adult brain, and both LPA and S1P are generated by neurons [9-11]. The biological consequences of lysophospholipid signaling in the nervous system are incompletely defined, but evidence for several roles in neural progenitors is emerging. As discussed above, there are clear roles for S1P and LPA in early neural tube development. Further, LPA appears to regulate cortical neurogenesis by promoting morphological changes, survival, and differentiation [12,13]. Finally, S1P activity is implicated in mediating migration of neural progenitor cells toward sites of spinal injury [10]. Thus, LPA and S1P regulate critical responses in neural progenitor cells that may be exploited to manipulate these cells in traditional pharmacological or cell-based therapeutics.

LPA and S1P bind and activate cell surface G-protein coupled receptors (GPCRs) to regulate cell proliferation, differentiation, and morphological changes, all of which may contribute to their roles in regulating neural progenitor cell function. There are at least five distinct LPA receptors (LPA1-LPA5) and five S1P receptors (S1P1-S1P5) [14]. LPA and S1P receptors couple to multiple G-protein pathways to regulate ion channel activity, adenylyl cyclase mediated cyclic AMP (cAMP) production, phospholipase C (PLC) mediated inositol phosphate production and calcium release, activation of the small GTPase Rho, and transactivation of receptor tyrosine kinase receptors (Review: [15]).

Regulation of cell growth and morphology are common effects of lysophospholipids. LPA and S1P have potent proliferative effects in multiple neural cell lines [16-18]. For example, LPA induces proliferation in neurospheres isolated from rat embryonic cortex [19], and application of S1P to neural progenitor cells from embryonic rat hippocampus has been shown to stimulate G_i/o _pathways which activate Mitogen-Activated Protein (MAP) kinases and DNA synthesis [20]. The latter observation is consistent with the mechanism for lysophospholipid stimulated proliferation in many cancer cells, in which LPA receptors transactivate the epidermal growth factor receptor (EGFR) pathway, resulting in MAP kinase activation and subsequent proliferation [16-18].

LPA and S1P also stimulate specific cytoskeletal rearrangements, likely contributing to their roles in axonal pathfinding and migration. Neural cell lines such as NIE-115 cells and PC12 cells undergo rapid and transient neurite retraction in response to LPA and S1P [21,22]. LPA induces neurite retraction within minutes, and neurons re-extend neurites after LPA is removed; thus, the retraction is dynamic and may fine tune neurite growth [23,24]. Similar neurite retraction and growth cone collapse occur in response to LPA in differentiating cortical neurons [23]. Morphological changes also occur in neural progenitor cells, which lack distinct neurites. Both LPA and S1P cause transient aggregation of rat hippocampal neural progenitor cells [20], and LPA stimulates cluster contraction, lamellipodia retraction and migration toward the center of the cluster in mouse cortical neuroblasts [11]. LPA stimulates cell rounding of cortical neural progenitors, important in cortical neurogenesis [11]. The mechanisms for these effects is incompletely understood, but in most cases LPA and S1P induced morphological changes can be partially or completely blocked by pretreatment with inhibitors of the small GTPase Rho or its primary effector in neurons, p160 Rho kinase (ROCK) [23,24].

The goal of the current study was to define functional lysophospholipid receptor signaling pathways in hES-NEP cells. We have determined that functional LPA and S1P receptors are expressed in hES-NEPs and regulate second messenger pathways, MAP kinase-dependent cell proliferation, and Rho-dependent morphology changes. These results contribute to the molecular characterization of hES-NEP cells, and establish for the first time a human, multipotent, renewable model cell system in which to define the role of LPA and S1P in neural progenitor cell function.

## Results

### LPA and S1P receptor mRNA transcript expression changes during the transition from ES cells to hES-NEP cells

**Figure 1:**
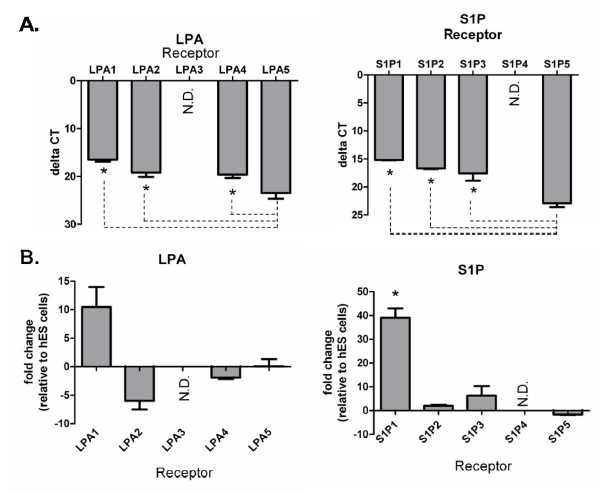
**LPA and S1P receptor subtype transcript expression in hES-NEP cells**. A Semi-quantitative RT-PCR from hNP cells revealed the relative expression of receptor mRNA. Higher expression is equivalent to a smaller delta CT value (CT value of RNA - CT value of 18s). Statistical significance was measured using a one-way analysis of variance (ANOVA) and Tukey's post-hoc comparisons were used to test the significance between all pairwise comparisons (p < .05). Data is represented as the mean ΔCT +/- S.E.M. (CT value of gene - CT value of 18 s) of 3 biological replicates. * in A demonstrates a significant difference (greater expression) than S1P5, * in B demonstrates a significant difference (greater expression) than LPA5. N.D. indicates that the average CT was >35 indicated that the mRNA signal was not detectable. B. Total RNA was isolated from WA09 hES cells and hES-NEP cells, and relative expression of each LPA and S1P receptor transcript was determined using quantitative RT-PCR. Results are reported as fold change in RNA transcript in hES-NEP cells relative to ES cells (details of data analysis in Methods). Data represents three compiled independent experiments and was subjected to ANOVA, tukey post-hoc analysis. Error bars represent standard error; *: p < 0.0001.

Expression of transcript encoding all five LPA receptors has been reported in hES cells and in hES cell-derived neurospheres [25], and three S1P receptors (S1P1-3) have also been detected in hES cells [26]. As described, the hES-NEP cell line used in this study was derived from the hES cell line, WA09. We performed quantitative RT-PCR to determine expression of transcript of LPA and S1P receptor subtypes in hES-NEP cells, and to determine if receptor expression changed in the transition from embryonic stem cell line to neural epithelial cell line. WA09 ES cells had detectable levels of transcript for all five LPA receptor genes and all five S1P receptor genes; however, in the hES-NEP population LPA3 and S1P4 were not expressed at detectable levels after 40 amplifications. Because the RT-PCR primer pairs used have been shown to have equivalent amplification efficiency (100% +/- 10%) at the annealing temperatures used, the relative expression of LPA and S1P receptors can be directly compared within hES-NEP cell RNA. The ΔCT value for each receptor transcript was determined by normalizing with CT values for the endogenous 18s ribosomal RNA. As shown in Figure 1A, LPA5 receptor transcript expression was significantly lower than LPA1, 2, and 4. Similarly, S1P 1, 2, and 3 transcripts were expressed at significantly higher levels in hES-NEP cells than S1P5. We further determined the fold change in transcript expression of LPA1, 2, 4, and 5 and S1P 1, 2, 3, and 5 in hES-NEP cells relative to their expression in the parent ES cell line WA09. LPA1 receptor transcript expression was increased approximately ten fold while LPA2 expression was decreased approximately five fold in cumulative data representing three experiments, but these changes did not meet criteria for statistical significance. Expression of LPA4 and 5 mRNA transcripts were relatively unchanged between the two populations. S1P1 receptor transcript was dramatically upregulated approximately forty fold in hES-NEP cells relative to the parent ES cell line (Figure 1B), while significant changes were not observed in expression of S1P 2, 3, and 5 transcript.

### NEP cells express functional LPA and S1P receptors

**Figure 2:**
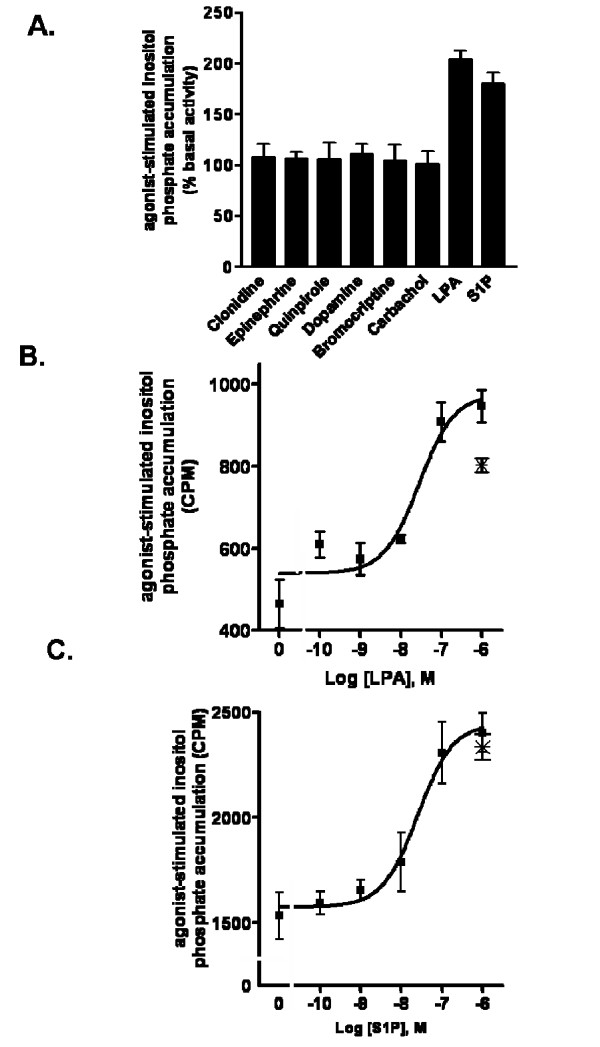
**hES-NEP cells express functional LPA and S1P receptors coupled to PLC**. (A) hES-NEP cells were treated with each of the indicated drugs at 10 μM, except for LPA which was assessed at 1 μM, for 30 minutes and assayed for IP levels as described in Methods. Results are reported as percent of basal inositol phosphate accumulation (CPM) with counts for drug treated wells divided by data from vehicle treated wells. (B) hES-NEP cells were treated with various concentrations of LPA in the presence (star) or absence (square) of Ptx for 30' and assayed for IP production. (C) hES-NEP cells were treated with various concentrations of S1P in the presence (star) or absence (square) of Ptx for 30' and assayed for IP production. Data are consistent with three independent experiments.

To evaluate expression of GPCRs for LPA and S1P as well as major neurotransmitter classes in hES-NEP cells, we screened agonists of adrenergic, dopamine, muscarinic acetylcholine, LPA, and S1P receptors for activity in assays measuring second messenger production. First, we assessed activity of these compounds in inositol phosphate assays that measure PLC activity. Cells were stimulated with each of the following drugs at a concentration of 10 μM for 30 minutes: clonidine (α_2 _adrenergic receptor agonist), epinephrine (general adrenergic receptor agonist), quinpirole (D2-like dopamine receptor agonist), bromocriptine (D2-like dopamine receptor agonist), carbachol (general muscarinic acetylcholine receptor agonist), and S1P (general S1P receptor agonist); 18:1 (Oleoyl) LPA (general LPA receptor agonist) was tested at a concentration of 1 μM due to loss of activity at higher concentrations. At these concentrations, only LPA and S1P stimulated a significant increase in inositol phosphate accumulation compared to vehicle treatment in hES-NEP cells (Figure 2A). We then generated LPA and S1P dose-response curves in these cells. The EC_50 _for inositol phosphate accumulation stimulated by either LPA or S1P is approximately 25 nM (Figure 2B, C). Pre-incubation with 100 ng/mL of the G_i/o _selective inhibitor Pertussis toxin (Ptx) for 18 hours did not inhibit S1P stimulated IP accumulation, indicating that this effect is not mediated by G_i/o _G-proteins, while Ptx consistently inhibited 30–40% of the LPA stimulated IP accumulation (✴, Figure 2B, C).

**Figure 3:**
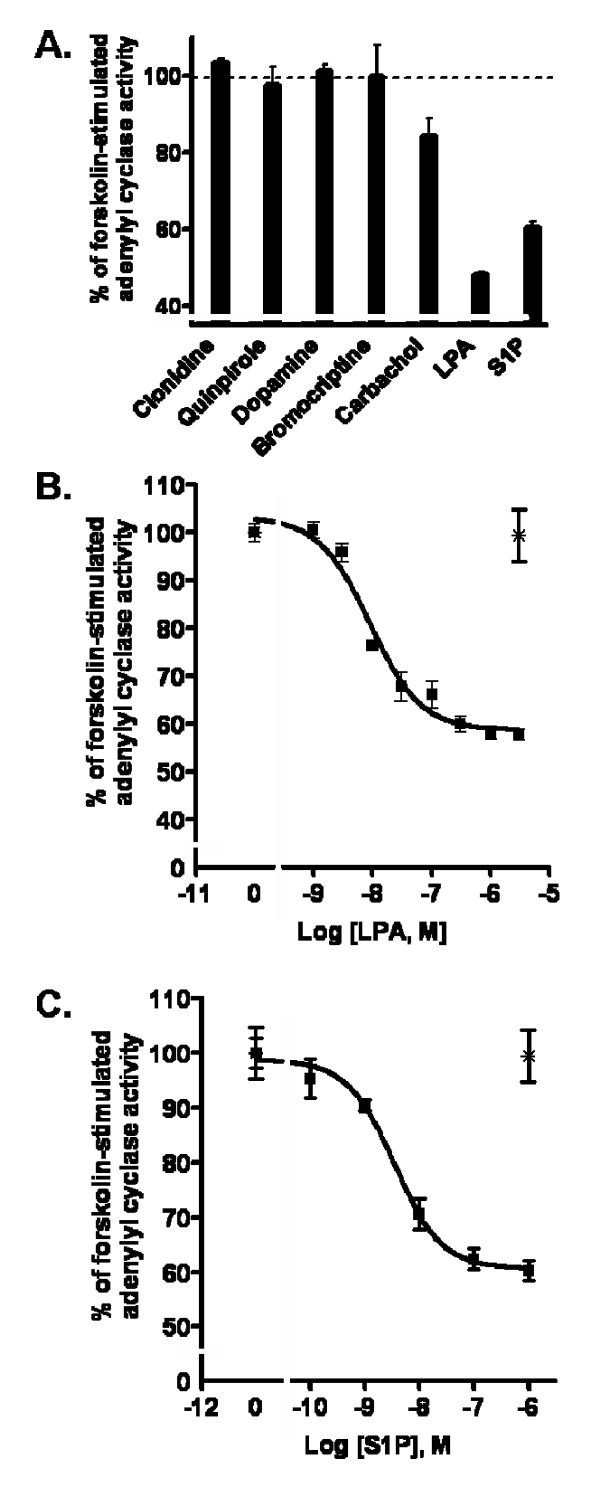
**hES-NEP cells express functional G_i/o_-coupled LPA and S1P receptors**. (A) hES-NEP cells were treated with 50 μM forskolin and 10 μM of each of the indicated drugs for 20', then assayed for cAMP levels as described in Materials and Methods. hES-NEP cells were treated with 50 μM forskolin and various concentrations of LPA (B) or S1P (C) for 20' in the presence (star) or absence (square) of Ptx. Data are consistent with three independent experiments.

We next determined if hES-NEP cells express functional adrenergic, dopamine, or lysophospholipid receptors coupled to G_s_-like increases in cAMP production. hES-NEP cells were treated with the same panel of agonist compounds (although quinpirole, bromocriptine, and carbachol do not activate any known G_s _coupled receptors), and none produced a significant increase in cAMP, suggesting there are not functional G_s _coupled LPA, S1P, adrenergic, or dopaminergic receptors expressed in hES-NEP cells (data not shown). Finally, the receptor agonists were added to cells following activation of adenylyl cyclase with forskolin to determine if they could decrease cAMP production via G_i/o _mediated inhibition of adenylyl cyclase. Adrenergic and dopaminergic receptor agonists had no effect on forskolin-stimulated cAMP levels, and carbachol produced a modest inhibition of cAMP production. In contrast, both LPA and S1P significantly inhibited forskolin-stimulated cAMP accumulation by approximately 50% and 40%, respectively, at 10 μM doses (Figure 3A). Dose response curves demonstrated that LPA inhibited forskolin-stimulated cAMP accumulation with an EC_50 _of approximately 10 nM (Figure 3B), while S1P had an EC_50 _of approximately 5 nM (Figure 3C). The activity of both LPA and S1P was completely inhibited by pre-incubation of cells with 100 ng/mL Ptx (✴, Figure 3B, C), confirming that this effect is mediated by G_i/o _G-proteins.

### LPA and S1P promote growth of hES-NEP cells via Ptx-sensitive G-proteins, EGF receptors, and MAP kinases

**Figure 4:**
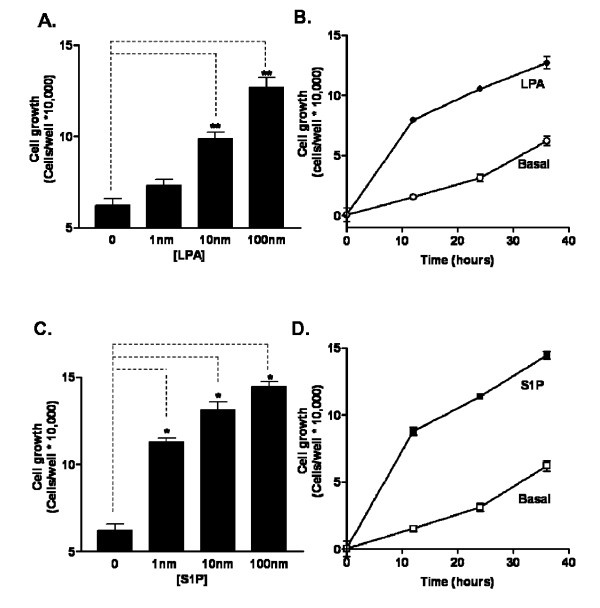
**LPA and S1P promote growth of hES-NEP cells**. (A, C) hES-NEP cells were plated in 24-well plates and grown to 50% confluence, and the treated with vehicle or 1–100 nM LPA (A) or S1P (C) for 36 hours and then counted. (B, D) Cells were incubated with vehicle or 100 nM LPA (B) or 100 nM S1P (D) and cell growth was determined at 12, 24, and 36 hours. Viable cells were counted and reported as the number of new cells/well. The number of cells at time zero (108,000) has been subtracted from all data shown. Data are representative of two independent experiments analyzed using an unpaired 2-tailed t-test. *: p < 0.05; **: p < 0.01.

To examine the effects of S1P and LPA on cellular growth, we determined the ability of LPA and S1P to stimulate growth of cultured hES-NEP cells over a 36 hour period by determining increases in cell number (Figure 4). hES-NEP cells were plated in 24-well plates and grown to 50% confluence. Cells were then grown for 36 hours with vehicle, 1 nM, 10 nM, or 100 nM LPA or S1P added to the normal growth media. Cells were not subjected to starve conditions, and therefore continued to grow at a normal basal rate in the absence of added lysophospholipid. Cells under basal growth conditions showed a 60% increase in cell number (increased to 170,000 cells/well at 36 hours, from 108,000 cells/well at time zero). Addition of lysophospholipid resulted in a dose-dependent increase in cell growth from 1 nM to 100 nM LPA (Figure 4A) and from 1 nM to 100 nM S1P (Figure 4B), with S1P showing an apparent higher potency. Cells treated with 100 nM LPA showed a 120% increase in cell number after 36 hours (235,000 cells at 36 hours), and cells treated with 100 nM of S1P showed a similar 130% increase in cell number (252,000 cells at 36 hours), as compared to the 60% increase in control cells. The basal growth rate was approximately linear over the 36 hour experiment (4B), and this rate was increased significantly by addition of 100 nM of either LPA (4B) or S1P (4D) as early as 12 hours. The rate of growth of LPA and S1P treated cells slowed at later time points as these cells approached confluency.

**Figure 5:**
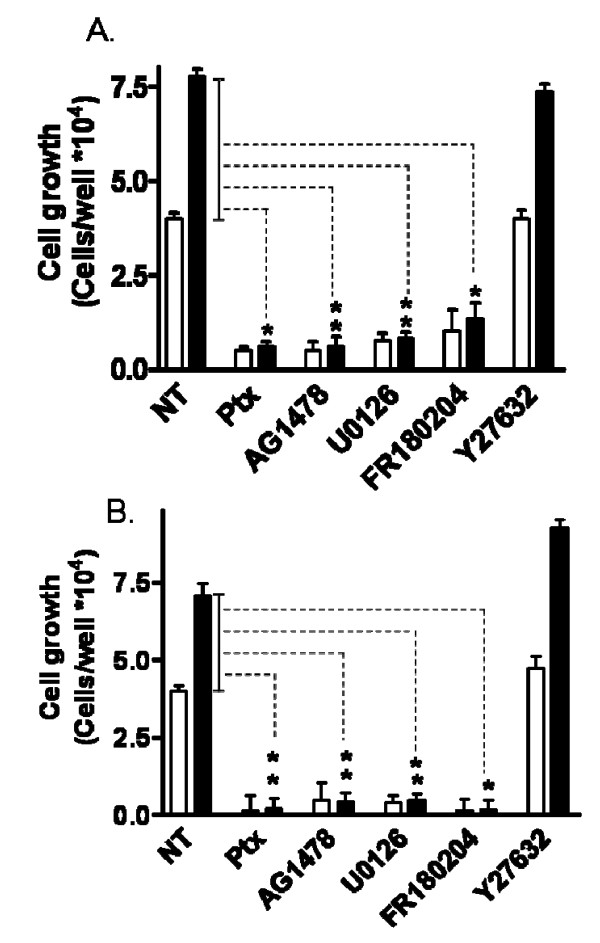
**LPA and S1P effects of hES-NEP cell growth are mediated by Ptx sensitive G-proteins, EGF receptors, and ERK Map kinases**. hES-NEP cells were pre-treated with 100 ng/mL Ptx, 2.5 μM AG1478, 10 μM U0126, 10 μM FR180204, 10 μM Y27632 or no treatment (NT) overnight and then treated with 100 nM LPA (A), 100 nM S1P (B) or vehicle. Cells were counted after treatment with inhibitors (time zero) and again after incubation with LPA or S1P. Results are reported as the number of new cells per well after LPA or S1P treatment; cell numbers at time zero have been subtracted. Data are representative of two independent experiments analyzed using an unpaired 2-tailed t-test. Comparisons were made between the LPA stimulated fold increase over basal growth in each condition. *: p < 0.05; **: p < 0.01.

MAP kinases such as p44 and p42 Extracellular signal Regulated Kinases (ERKs) are known to play an important role in neural progenitor cell proliferation [27-29], and both LPA and S1P activate the MAP kinase pathway in multiple systems [20,30-37]. Further, LPA has been shown to activate MAP kinase pathways through a G_i/o_-dependent EGF receptor transactivation mechanism [16-18]. To determine which of these pathways is functional in lysophospholipid stimulated growth of hES-NEP cells, the effects of pretreatment with specific pharmacological inhibitors of pathway intermediates were determined: the G_i/o _selective inhibitor Ptx (100 ng/mL), the EGF receptor inhibitor AG1478 (2.5 μM), the MAP kinase/ERK Kinase (MEK) inhibitor U0126 (10 μM), the direct ERK inhibitor FR180204 (10 μM), and the p160ROCK inhibitor Y27632 (10 μM). Cells were counted after pre-treatment with inhibitor and again after an 18 hour incubation with LPA (Figure 5B) or S1P (Figure 5D). Both LPA and S1P significantly induced increased cell growth over vehicle at this time point. Pre-treatment with Ptx, AG1478, U0126, and FR180204 completely inhibited both basal cell growth and LPA and S1P stimulated growth; however, the p160ROCK inhibitor Y27632 did not significantly affect basal growth or growth stimulated by either LPA or S1P. Further, pre-treatment with the inhibitors did not increase cell staining with Trypan Blue, indicating that these compounds were not cytotoxic at the concentrations used (data not shown). These results suggest that LPA and S1P promote growth of hES-NEP cells through a mechanism dependent on Ptx-sensitive G_i/o _G-proteins, EGF receptor, MEK, and ERK, but independent of the Rho-associated kinase p160ROCK.

**Figure 6:**
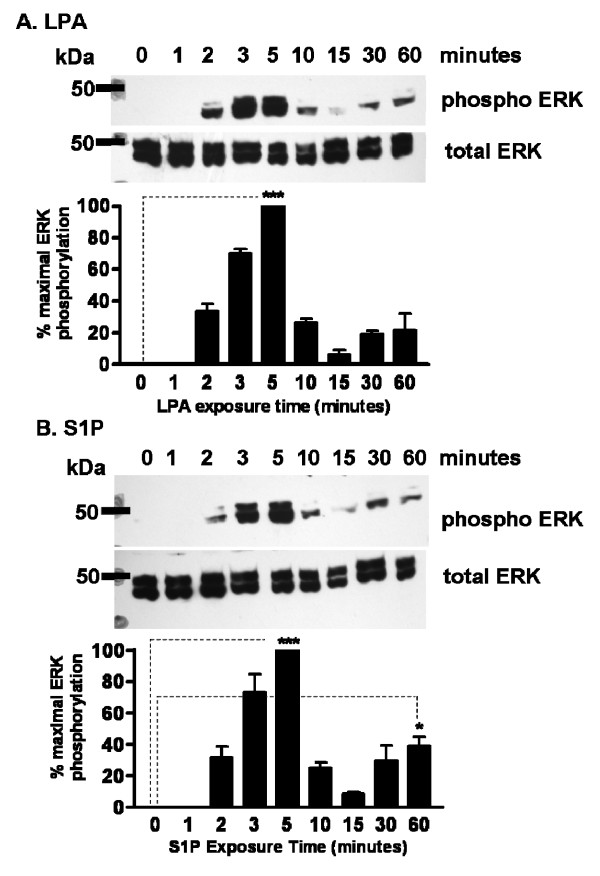
**LPA and S1P induce ERK phosphorylation in hES-NEP cells**. hES-NEP cells were treated with 1 μM LPA (A) or 100 nM S1P (B) for the indicated amounts of time and then assayed for phosphorylated p44/42 ERK and total p44/42 ERK as described in Materials and Methods. In each case, results are shown as a Western blot image of a representative experiment and a densitometry graph of combined data from two independent experiments analyzed using an unpaired 2-tailed t-test *: p < 0.05; **: p < 0.01; ***: p < 0.001.

The data above implicate MAP kinase activation in the ability of LPA and S1P to stimulate cell growth. Thus, we directly tested the ability of LPA and S1P to stimulate phosphorylation of the MAP kinase proteins p44/42 ERK. We performed Western blotting on cellular lysates after treating cells with either 1 μM LPA or 100 nM S1P for time points between one and sixty minutes. LPA and S1P each stimulated p44/42 ERK phosphorylation relative to total p44/42 ERK protein, with peak phosphorylation occurring after 5 minutes of stimulation, followed by a later sustained lower level of phosphorylation at 30–60 minutes (Figure 6). The latter peak was consistently observed in both LPA and S1P treated cells, but did not meet statistical criteria for significance in LPA treated cells.

### LPA and S1P induce reversible morphological changes in hES-NEP cells

**Figure 7:**
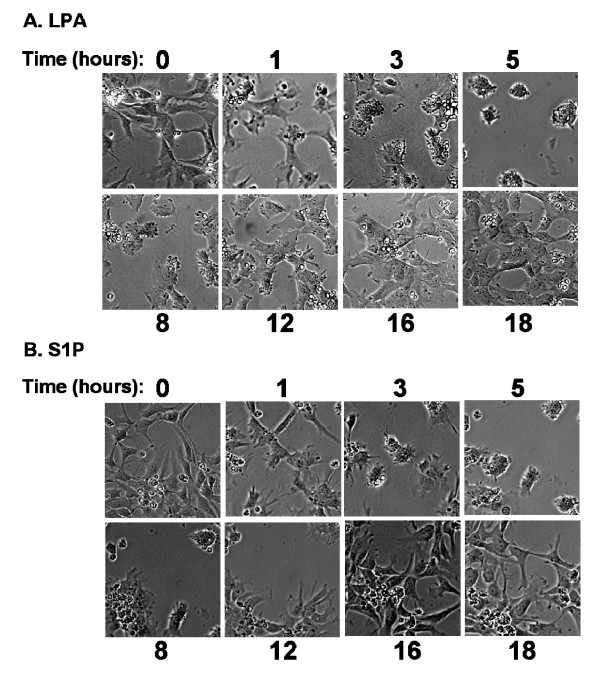
**LPA and S1P induce reversible morphology changes in hES-NEP cells**. hES-NEP cells were incubated with (A) 1 μM LPA or (B) 100 nM S1P for 18 hours and subjected to continuous video microscopy. Still images of cell morphology changes were recorded as described in Materials and Methods at select time points. Data images are consistent with three independent experiments.

**Figure 8:**
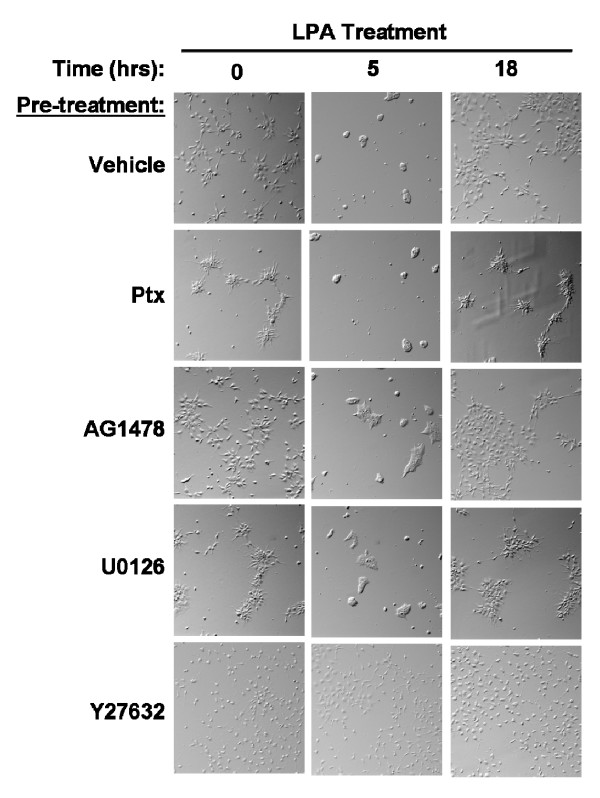
**Morphology changes induced by LPA are blocked the p160ROCK inhibitor Y27632.** hES-NEP cells were incubated with vehicle, 100 ng/mL Ptx, 2.5 μM AG1478, 10 μM U0126, or 10 μM Y27632 for 18 hours and then treated with 1 μM LPA for 18 hours. Images of cell morphology were captured  after treatment with each inhibitor but before addition of LPA (t =  0), after five hours of LPA treatment (t = 5) and after 18 hours of LPA treatment (t = 18). Data images are consistent with three  independent experiments.

**Figure 9:**
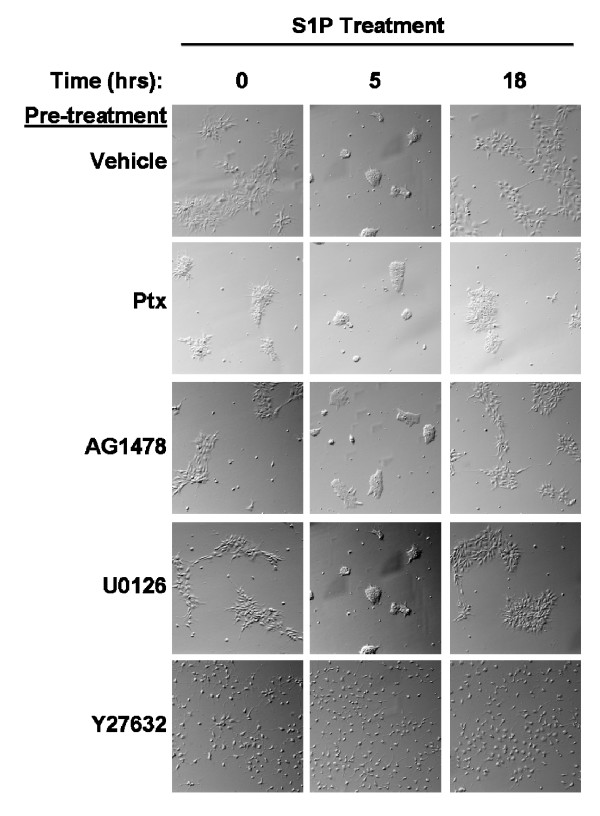
**Morphology changes induced by S1P are blocked the p160ROCK inhibitor Y27632.** hES-NEP cells were incubated with vehicle, 100 ng/mL Ptx, 2.5 μM AG1478, 10 μM U0126, or 10 μM Y27632 for 18 hours and then treated  with 100 nM S1P for 18 hours. Images of cell morphology were captured after treatment with each inhibitor but before addition of S1P (t = 0), after five hours of S1P treatment (t = 5) and after 18 hours of S1P treatment (t = 18). Data images are consistent with three independent experiments.

LPA and S1P mediate morphological changes reflecting cytoskeletal rearrangements in multiple neuronal cell types. We determined the effect of LPA and S1P on hES-NEP cell morphology using continuous live cell microscopy. hES-NEP cells were plated and maintained in an environmentally controlled slide incubator system that allows continuous video surveillance of live cells under controlled temperature and atmospheric conditions. After treatment with 1 μM LPA (Figure 7A) or 100 nM S1P (Figure 7B), hES-NEP cells became aggregated and rounded, retracting cellular extensions. This morphological change was transient, reaching a peak at approximately 5 hours after treatment and returning to baseline 18 hours after treatment (Video of the complete experiment available in: Additional files 1 and 2). Addition of vehicle caused no morphological changes under these conditions (data not shown). In contrast to the effects on the proliferative response, overnight pre-treatment of the cells with Ptx, AG1478, or U0126 did not block the ability of LPA (Figure 8A) or S1P (Figure 9) to induce morphological changes, while pre-treatment with Y27632, the inhibitor of p160ROCK, completely prevented cellular aggregation and rounding induced by either lysophospholipid. These data suggest that morphological changes induced by LPA and S1P are mediated by a pathway that does not include G_i/o _proteins, EGF receptors, or MEK, but does require the Rho effector p160 ROCK. Notably, Ptx treatment alone caused some cellular aggregation; however, treatment with either LPA or S1P induced further cell rounding. Further, cells pre-treated with Y27632 had longer, thinner membrane extensions than cells pre-treated with vehicle, consistent with previous observations by Darenfed et al. [38].

## Discussion

Lysophospholipids are hypothesized to be critical regulators of neuronal differentiation, proliferation, and migration during development and following injury. While rodent neural progenitor cells and human transformed cell lines have been used to establish these roles and investigate the pathways responsible, the effects of lysophospholipids in human neural progenitor cells has not been established until now. This study establishes our recently characterized human embryonic neural epithelial progenitor cell line as a valid model system to define the role of LPA and S1P in neural progenitors during human neural development, differentiation, and wound healing.

Our results demonstrate that hES-NEP cells express functional LPA and S1P receptors coupled to G_i/o _mediated inhibition of adenylyl cyclase and to a pertussis toxin-insensitive PLC pathway, likely mediated by G_q_. hES-NEP cells do not express functional G_s _coupled receptors for either LPA or S1P. Like the cAMP inhibitory response, the proliferative response was also completely inhibited by Pertussis toxin (Ptx) and is therefore also mediated by G_i/o _coupled receptor subtypes. In contrast, the morphological response was not inhibited by Ptx, and so is not mediated by G_i/o _coupled receptors. Our data suggest that LPA and S1P morphological responses may be mediated by G_12 _coupled GPCRs, consistent with the observed Rho-dependency, although we cannot rule out a G_q_-mediated mechanism. All LPA and S1P receptors except LPA3 and S1P4 were detected in hES-NEP cells. Studies including additional pharmacologically selective drugs are required to determine the molecular identity of the receptors mediating the observed responses in hES-NEP cells.

Both LPA and S1P stimulate proliferation of many cell types. Studies in multiple cell lines [16-18,39,40] suggest that LPA receptors coupled to G_i/o _stimulate cell growth via EGF receptor transactivation and subsequent MAP kinase activation, which directly leads to cell proliferation. While we observed a strong effect of lysophospholipids on cell growth, our data do not distinguish between effects on proliferation versus survival pathways. Future work should directly address the effect of LPA and S1P on apoptosis in these cells. Indeed, LPA does function as a survival factor in many cancer cell types via activation of the PI3 Kinase pathway. Nonetheless, our data are consistent with the proliferative EGF receptor transactivation mechanism described above. The growth responses to LPA and S1P in these cells were completely inhibited by Ptx and inhibitors of EGF receptors and ERK Map kinases, but not by inhibitors of p160 ROCK. Notably, the basal growth of hES-NEP cells was also inhibited by EGFR and MAP kinase inhibitors but not p160 ROCK inhibitor, suggesting that basal growth is mediated by a similar pathway, although not necessarily initiated by LPA or S1P. This also suggests a basal level of ERK MAP kinase activity. Although the data shown in Figure 6 do not show basal ERK phosphorylation due to the short exposure times required to avoid saturation of peak bands for quantification, in longer exposures basal ERK phosphorylation was apparent (data not shown).

The proliferative effect of LPA has been directly demonstrated in rat embryonic neural stem cells [19]. Cui et al. report a bell-shaped LPA dose response relationship in proliferation assays in which LPA increased thymidine incorporation at concentrations between 10 nanomolar and 1 micromolar, but inhibited proliferation at higher concentrations. This biphasic effect of LPA on proliferation is consistent with both our observation that LPA stimulates hES-NEP cell growth between 1 nM and 100 nM, and a recent report in which 10 micromolar LPA did not stimulate proliferation in human neurospheres [25]. Similarly, LPA stimulated production of inositol phosphates reached a maximal level at 1 μM and a reduced activation at higher concentrations.

LPA and S1P effects on morphology of either neurons or neural progenitors are mediated by effects on the actin cytoskeleton and/or microtubules, and effects are typically, but not always, dependent on the small GTPase protein Rho. Rho is known to regulate axonal growth, neuronal differentiation, and neuronal survival, primarily through its well-characterized neuronal effector p160 ROCK (Review:[41]). Rho activation occurs primarily via activation of Rho exchange factors by G proteins of the G_12 _subfamily, and leads to activation of p160 ROCK which mediates morphological changes by altering cytoskeletal structure. Specifically, p160 ROCK increases actin contractility and stress fiber formation via myosin-II regulatory light chain (MLC) [41] and decreases actin depolymerization via LIM kinases to regulate growth cone collapse [24]. Alternately, G_i/o _pathways can also alter the cytoskeleton through activation of Glycogen synthase kinase-3 (GSK-3) [42] or Rac, which promotes cell spreading [43,44].

The effect of LPA on neural cell morphology varies with cell type and distinct morphology changes occur over different time scales. Typically, in neurons or neuronal cell lines that have neurites or growth cones, these retract and cells round in response to LPA within minutes. In NIE-115 and NG108-15 cells, and B103 cells expressing either LPA1 or LPA4, LPA causes a rapid, transient rounding which initiates at 5 minutes following LPA addition, and cells recover their flattened morphology after 20 minutes, even in the continued presence of LPA [21,44]. Alternately, in rat hippocampal NP cells both LPA and S1P cause transient aggregation with a maximal response at 3 hours and a return to baseline at 18 hours [20,44]. Similarly, in B103 cells expressing exogenous LPA4, but not LPA1, LPA stimulated a slow aggregation that peaked at three hours [44]. Like the rapid cell rounding, the slow cell aggregation response is dependent on the Rho effector p160 ROCK, as was the slow cell aggregation observed in this report. In contrast, the known activation time course of p160 Rho kinase is on a scale of minutes, and Rho activation occurs even faster. Thus, even though this response is dependent on Rho/Rho kinase activation, these are not the rate limiting factors in the response. In our experiments, LPA or S1P were added to the media and not washed out throughout the experiment. The long recovery time of shape changes may reflect time course of LPA stability in the media. Consistent with this explanation, when media was changed to remove S1P one hour after addition to cells, morphology changes immediately began to reverse.

Our data clearly implicate Rho-mediated activation of ROCK in mediating LPA and S1P stimulated rounding and aggregation in hES-NEP cells. Inhibition of p160 ROCK completely blocked LPA and S1P stimulated effects, while both phospholipids could still mediate cell aggregation and rounding following inactivation of EGFR, or ERK. Although LPA and S1P still clearly altered cell morphology following treatment with Ptx, Ptx treatment itself induced modest cell aggregation. This effect of Ptx may reflect inhibition of basal G_i/o _mediated effects on GSK-3 or Rac as described above.

While the current study describes LPA and S1P effects on proliferation and morphological changes, hES-NEPs are also a promising model cell system in which to study LPA and S1P effects in multiple processes of neural development. There is growing evidence that S1P and LPA regulate neuronal differentiation; however, data from various models report contradictory effects [11,19,20,25,26]. For example, LPA is reported to increase neuronal differentiation of rat neural progenitors (NP) [19] and mouse neurosphere cultures [11], while more recently LPA was shown to inhibit neuronal differentiation of human ES cell-derived neurosphere cultures [25]. These contradictions may reflect bona fide differences in LPA signaling pathways in rodent versus human neural differentiation, or they may be a result of mixed cell populations and the various sources and developmental stages from which the neural stem cells were isolated. For example, significant differences in expression of FGF, wnt and LIF pathway genes are observed between human neural stem cells derived from hES cells and fetal neural stem cells [45]. Given these potential differences between neural stem cells from different cell sources, homogeneous multi-potent human ES cell-derived neuroepithelial (hES-NEP) cells may be a superior model system in which to elucidate the roles of LPA and S1P cell signaling pathways in neural progenitor cells. Future studies of LPA and S1P effects on differentiation in the homogenous hES-NEP cell system will serve to clarify the effect of lysophospholipids on human neural differentiation.

## Conclusion

We have defined LPA and S1P signaling pathways in hES-NEP cells that promote cellular growth and morphological changes by distinct mechanisms. This cell system is superior to rodent and transformed cell systems in which LPA and S1P effects have been defined by virtue of its human origin, multi-potent status, and non-transformed state. Further, as a stable, homogeneous, adherent, renewable cell line, hES-NEP cells are a convenient model system for future studies defining the functional role of lysophospholipids in proliferation, differentiation, and migration in the developmentally important human neural progenitor cell type.

## Methods

### Materials

Carbachol, epinephrine, quinpirole, clonidine, bromocriptine, dopamine, and U0126 were purchased from Sigma-Aldrich (St. Louis, MO). Y27632 and AG1478 were purchased from Tocris Bioscience (Ellisville, Missouri). Pertussis toxin was purchased from List Biological Laboratories (Campbell, CA) and FR180204 from EMD Biosciences (La Jolla, CA). Oleoyl (18:1) LPA and D-erythro-sphingosine-1-phosphate were from Avanti Polar Lipids (Alabaster, AL).

### Cell Culture

Commercially available stocks of hES-NEP cells [available as ENStem-A™ (Millipore, Temecula, CA)] were used. These cells were derived from WA09 human ES cells and maintained as described previously[1]. Briefly, cells were grown on poly-ornithine (20 μg/mL)/laminin (5 μg/mL) (Sigma-Aldrich St. Louis, MO) coated plates in ENStem-A™ Neural Expansion Medium with 2 mM L-Glutamine and 20 ng/mL b-FGF (all from Millipore, Temecula, CA). Cells were passaged approximately every 48 hours and split 1:2 following manual dissociation by trituration.

WA09 (WiCel) were cultured in Dulbecco's minimal essential medium/Ham's F12 medium (DMEM/F12), 2 m*M *L-glutamine, 0.1 m*M *minimal essential medium (MEM) nonessential amino acids, 50 U/ml penicillin, 50 μg/ml streptomycin (all from Invitrogen), 4 ng/ml basic fibroblast growth factor (bFGF; R&D Systems, Minneapolis, MN) and 20% KSR (Invitrogen). Cells were cultured on mitomycin-C (Sigma Chemical Co., St. Louis, MO) mitotically inactivated murine embryonic fibroblasts, manually dissociated, and passaged to new feeder layers every 4–5 days [46].

### Real Time Reverse Transcriptase PCR

RNA was extracted using Qiashredder and RNeasy kits (Qiagen, Valencia, CA) according to the manufacturer's instructions. The RNA quality and quantity was verified using a RNA 600 Nano Assay and an Agilent 2100 Bioanalyzer (Agilent Technologies, Santa Clara, CA). Total RNA was reverse-transcribed using the cDNA Archive Kit (Applied Biosystems Inc., Foster City, CA) according to manufacturer's protocols. Quantitative RT-PCR (Taqman) assays were chosen for the transcripts from a pre-validated library of human specific QPCR assays, and incorporated into a 384-well Micro-Fluidics Cards. Relative quantification was carried out on the ABI PRISM 7900 Sequence Detection System (Applied Biosystems Inc., Foster City, CA). Expression data for each LPA or S1P receptor was first normalized against endogenous 18S ribosomal RNA within each cDNA, and then the relative expression in hES-NEP was compared to hES cells using the ΔΔCT method of quantification in SDS software (Applied BIosystems Inc., Foster City, CA). Relative fold changes were determined as RQ values for positive changes and -1/RQ values for negative fold changes. ANOVA statistical analysis was performed using Tukey post-hoc analysis.

### Inositol Phosphate Assay

Production of Inositol Phosphates (IP) was quantified using established protocols [47]. Briefly: To measure IP production by PLC activation, hES-NEP cells were plated in 24-well dishes at ~80% confluency. Cells were labeled with 1 μCi/well [^3^H] myo-inositol (American Radiolabeled Chemicals, St. Louis, MO) for 18 hours to label the cellular pool of phosphatidyl inositol. The cells were treated with Oleoyl (18:1) LPA (Avanti Polar Lipids, Alabaster, AL) or D-erythro-sphingosine-1-phosphate (Avanti Polar Lipids, Alabaster, AL) in the presence of 10 mM lithium chloride to inhibit degradation of inositol phosphates for 30 minutes at 37°C. Cells were then lysed in cold formic acid and neutralized with ammonium hydroxide, and the lysates were then loaded onto columns of AG 1-X8 anion exchange resin (Biorad, Hercules, California). The columns were washed with water and dilute ammonium formate to remove unhydrolyzed lipids. The [^3^H] IPs were then eluted with 1.2 M ammonium formate/0.1 M formic acid, and added to scintillation cocktail for counting. In some experiments, cells were treated with 100 ng/mL pertussis toxin (Ptx) for 18 hours prior to IP assay.

### cAMP Assay

We used a modified version established protocols [48]. hES-NEP cells were plated in 12-well dishes and labeled with 0.6 μCi [^3^H]-adenine (Perkin Elmer, Waltham, MA) for three hours in the presence or absence of 200 ng/mL Ptx. Assay buffer containing 1 mM isobutylmethylxanthine (IBMX), 50 μM forskolin, and varying concentrations of LPA was added to the cells for 20 minutes at 37°C. Reactions were terminated by aspiration followed by the addition of stop solution containing 1.3 mM cAMP and 2% sodium dodecyl sulfate. [^14^C]-cAMP stock was added to each well to control for recovery of cAMP, followed by perchloric acid to lyse cells. Lysates were neutralized with KOH and cAMP was isolated using sequential column chromatography over Dowex AG-50-W4 cationic exchange resin (Bio-Rad, Hercules, CA) followed by neutral alumina columns. The resulting eluate was subjected to scintillation counting after the addition of scintillation cocktail.

### Cellular Growth

hES-NEP cells were plated in 24-well plates at 50,000 cells per well and grown to reach 50% confluency (approximately 100,000 cells/well). In some experiments, cells were pre-treated with the indicated reagents for 18 hours, triturated to remove them from the plate, and counted using a hemacytometer to determine the number of cells per well. Cells were then treated with LPA, S1P, or vehicle for the indicated amount of time and counted again. Trypan blue exclusion was used to determine cell viability following drug treatment (0.4% (wt/vol) solution of Trypan Blue (Invitrogen, Carlsbad, CA)). Statistical significance of changes in growth was determined using an unpaired, two-tailed T-test.

### p44/42 ERK MAP Kinase Phosphorylation

hES-NEP cells were plated in 24-well plates. Prior to the assay, cells were washed one time with ENStem-A™ Neural Expansion Media and allowed to incubate in 250 μL media for 15 minutes at 37°C. LPA or S1P was then applied to the cells for the indicated period of time. The reaction was terminated by aspirating the media and adding 100 μL protein sample buffer. Cells were harvested and lysed in protein sample buffer, separated by SDS-PAGE, transferred to nitrocellulose membranes, and immunoblotted using a primary antibody targeted against phospho-ERK or total ERK (Cell Signaling Technology, Danvers, MA) and peroxidase-conjugated secondary antibodies (Bethyl Laboratories, Montgomery, TX). Bands were then visualized using SuperSignal Chemiluminescent substrate (Pierce, Rockford, IL). Densitometry analysis was performed using Total Lab 1D Gel Analysis software. Background bands were not subtracted out and all lanes and bandwidths were of equal size. Densitometry results for phospho-ERK were normalized to total ERK to control for loading, and then normalized to maximal ERK phosphorylation to compare between experiments. Statistical significance of increases in ERK phosphorylation over basal levels was determined using an unpaired, two-tailed T-test.

### Cell Morphology Studies

Continuous video microscopy of hES-NEP cells was performed using the WaferGen Smart Slide System (WaferGen, Incorporated, Freemont, CA). hES-NEP cells were plated on a WaferGen Smart Slide 100 and maintained at 37°C, with the lid at 39°C to prevent condensation. CO_2 _was maintained at 5% over the course of the experiment, and negative flow was maintained through systemic purging every two minutes. Images were obtained using a Nikon Eclipse TE2000-S microscope, and captured every two minutes using a Retiga 2000R Fast 1394 camera (QImaging, Canada). Data were processed using Image Pro Plus5.1 version 5.1.0.20 (Media Cybernetics, Inc., Bethesda, MD).

To study the effects of pharmacological inhibitors on LPA and S1P stimulated changes in morphology, hES-NEP cells were plated in 6-well plates. Three areas with approximately equal cell densities were identified in each well and an image of each of these areas was captured with a Nikon AZ100 microscope mounted with a Nikon Digital Sight DS-QiMc camera set at 16× magnification. Cells were pre-treated with the indicated compounds for 18 hours. LPA or S1P was then applied for an additional 18 hours. Images of the cells were captured in triplicate after pre-treatment, approximately 5 hours after application of LPA or S1P, and then again 13 hours later.

## Authors' contributions

JHH carried out inositol phosphate and cAMP assays, cell growth assays, ERK phosphorylation assays, and morphology studies with inhibitors; JM performed video microscopy for morphology studies; DM and CS performed qRT-PCR assays; PC carried out inositol phosphate assays; SS and SBH conceived of the study, SBH was primarily responsible for drafting the manuscript, SS participated in key manuscript revisions, all authors participated in the final manuscript revision and approval.

## Supplementary Material

Additional file 1 Continuous video microscopy of LPA treated hES-NEP cells. hES-NEP cells were incubated with 1 μM LPA for 18 hours and subjected to continuous video microscopy and condensed to the attached 2 minute time-lapse movie file. Click here for file

Additional file 2 Continuous video microscopy of S1P treated hES-NEP cells. hES-NEP cells were incubated with 100 nM S1P for 18 hours and subjected to continuous video microscopy and condensed to the attached 2 minute time-lapse movie file. Click here for file
